# Elevated plasma p‐tau levels may be linked to reduced sleep spindles among older African Americans

**DOI:** 10.1002/alz.091414

**Published:** 2025-01-09

**Authors:** Payton G. White, Miray Budak, Alyssa Arnasalam, Mustafa Sheikh, Bernadette A. Fausto, Patricia Fitzgerald‐Bocarsly, Fanyu Hercules Tawe, Nicholas J. Ashton, Kaj Blennow, Henrik Zetterberg, Mark A. Gluck

**Affiliations:** ^1^ Rutgers University–Newark, Newark, NJ USA; ^2^ Rutgers New Jersey Medical School, Newark, NJ USA; ^3^ Department of Psychiatry and Neurochemistry, Institute of Neuroscience and Physiology, The Sahlgrenska Academy, University of Gothenburg, Mölndal, Gothenburg Sweden; ^4^ Department of Psychiatry and Neurochemistry, Institute of Neuroscience and Physiology, The Sahlgrenska Academy, University of Gothenburg, Mölndal Sweden

## Abstract

**Background:**

Biomarkers, such as blood p‐tau181, p‐tau217, and p‐tau231, have been created and verified to mirror the pathophysiology of tau and amyloid‐β (Aβ) in the brain ^2^. Sleep spindles are known to contribute to memory consolidation and generalization and may therefore be a promising biomarker in preclinical Alzheimer’s Disease (AD) ^3,4,5^. The present study investigated the relationship between sleep spindles and p‐tau levels in cognitively healthy older African Americans.

**Method:**

Participants were drawn from the ongoing longitudinal study, Pathways to Healthy Aging in African Americans conducted at Rutgers University–Newark. 75 participants, ages 60‐87 years old (completed a blood draw and underwent at‐home sleep monitoring over two nights using the DREEM 3 Headband. Plasma was extracted from blood samples to assess p‐tau 231 levels using Single molecule array technology, measured by in‐house Simoa methods at the University of Gothenburg. Based on the sample median thresholds, a subset of participants were characterized as having higher p‐tau231 levels (> 8.9447 pg/mL).

**Result:**

Individuals with high levels of p‐tau 231 (> 8.9447 pg/mL) had significantly lower frontal sleep spindle relative power than individuals with lower p‐tau 231 levels (< 8.9447 pg/mL) (F= 1.110, η ^2^= 0.175, p= 0.378).

**Conclusion:**

Sleep spindle relative power may serve as a potential marker for p‐tau231 levels among cognitively unimpaired older African Americans in preclinical AD.

